# One Health approach for West Nile virus surveillance in the European Union: relevance of equine data for blood safety

**DOI:** 10.2807/1560-7917.ES.2019.24.16.1800349

**Published:** 2019-04-18

**Authors:** Johanna J Young, Denis Coulombier, Dragoslav Domanović, European Union West Nile fever working group, Hervé Zeller, Céline M Gossner

**Affiliations:** 1 European Centre for Disease Prevention and Control (ECDC), Stockholm; 2 Members of the European Union West Nile fever working group are listed at the end of the article

**Keywords:** West Nile fever, West Nile Virus, blood safety, One Health, zoonoses, European Union, blood-borne infections, equids, surveillance systems, risk assessment

## Abstract

West Nile virus (WNV) infection is notifiable in humans and equids in the European Union (EU). An area where a human case is detected is considered affected until the end of the mosquito transmission season (week 48) and blood safety measures have to be implemented. We used human and equine case notifications between 2013 and 2017 to define the WNV distribution in the EU and to investigate the relevance of using equine cases as a complementary trigger for blood safety measures. Adding areas with equine cases to the definition of an affected area would have a major impact on blood safety measures. Adding areas with equine cases where human cases have been reported in the past would increase the timeliness of blood safety measures with only a limited impact. Although the occurrence of human and/or equine cases confirms virus circulation in the EU, no evidence was found that occurrence of equine cases leads to human cases and vice versa. We conclude that information about equine data should contribute to raising awareness among public health experts and trigger enhanced surveillance. Further studies are required before extending the definition of affected areas to areas with human and/or equine cases.

## Introduction

West Nile fever (WNF) is a viral zoonotic disease that is considered a re-emerging public health challenge in the European Union (EU). Mosquitoes, primarily *Culex* genus, serve as vectors, and wild birds as reservoir hosts. Equids and humans are dead-end hosts [1]. In Europe, West Nile virus (WNV) transmission occurs primarily from April to November, when competent vectors are active and abundant. Most human infections are acquired via mosquito bites but transmission via blood transfusion, organ transplantation, in laboratory settings and from mother to fetus during pregnancy can occur [2]. The majority of human cases remain asymptomatic; ca 20% of infected individuals develop febrile illness and less than 1% develop severe neurological symptoms [3]. In comparison, 10% of the infected equids develop neurological symptoms with different levels of severity [4]. Although no vaccine is available for humans, a vaccine for equids has been available in the EU since 2008 [5].

At the EU level, WNV infection is notifiable for humans and equids [6,7]. One of the main goals of human WNV surveillance is to prevent human-to-human transmission via donation of contaminated substances of human origin (e.g. blood). Human cases are notified according to the EU case definition [8] by the national public health authorities through The European Surveillance System (TESSy) of the European Centre for Disease Prevention and Control (ECDC) [9].

Human WNV surveillance is mandatory in 22 EU countries [10]. Surveillance is comprehensive in most countries, meaning that it covers the whole population of the geographical area under surveillance (i.e. national, regional). Belgium, the Czech Republic, France, Greece, Slovakia and the United Kingdom have implemented active surveillance such as active case finding close to where the identified case lives or was exposed or active case finding in healthcare facilities or laboratories, while the remaining countries conduct passive surveillance.

Animal health authorities within the EU have to notify cases of equine encephalomyelitis due to WNV through the Animal Disease Notification System (ADNS) of the European Commission (EC) [7].

Equine surveillance is mostly passive. In Croatia, Greece, Romania and Spain, active equine surveillance is performed, including regular serological screening of sentinel horses.

According to the EU blood safety directive, blood establishments should defer donors for 28 days after leaving an affected area, defined as an area with ongoing transmission of WNV to humans, unless an individual donation nucleic acid test (NAT) is negative [2,11]. To support the implementation of the directive, since 2011, ECDC has been publishing weekly WNV epidemiological updates including the geographical distribution of human cases in the EU and neighbouring countries [12].

We present a descriptive epidemiological study based on the human and equine WNV infections notified at the EU level, and discuss the added value of using equine WNV infections as a complementary trigger for the implementation of blood safety measures in the EU.

## Methods

We extracted case-based data from TESSy and ADNS on human and equine WNV infections, respectively, for the 2013–17 period. The datasets were merged and the following variables were included in the analysis: host species (human or equid), place of infection (NUTS-3 level (nomenclature of territorial units for statistics)) [13] and time (week of disease onset). For 7% (56/847) of the human cases, we had to use alternative dates: date of notification (6%; 52/847) and date of diagnosis (0.5%; 4/847). An affected area is defined as a NUTS-3 level area with at least one laboratory-confirmed, autochthonous human WNV infection. When necessary, geo-coordinates were converted into NUTS-3 level. We included probable and confirmed human cases as well as confirmed equine cases [8]. Cases among humans who had been outside the country of notification during the incubation period were excluded. Human WNV infections in this study are not differentiated between non-neuroinvasive and neuroinvasive cases.

We used STATA/IC 13.0 (Stata Corp., College Station, TX, United States) statistical software and created maps using EMMa (ECDC, Solna, Sweden) [14].

We used 2016 and 2017 data to calculate the proportional increase in the impact of blood safety measures, if equine cases were to be used in addition to human cases for defining an affected area. We developed two alternative scenarios. In the first scenario, human case(s) or equine case(s) define an affected area and the first of these cases triggers the start of the season in that area. In the second scenario, in addition to human case(s), equine case(s) define an affected area if at least one human case has been detected in that area during the previous 3 years. This latter scenario takes into account areas in which a spill-over of the virus to humans has been previously identified.

We estimated the impact, in affected areas, on the size of the blood donor population subjected to blood safety measures for scenario 1 and 2 and calculated the percentage increase compared with the baseline impact.

We used as a baseline the sum of number of weeks (W) times the population in the area (P) for each affected area in the EU considering human cases only:

$\text{Impact Baseline}=\overset{n}{\underset{i=1}{\sum }}\left(W_{i}\times P_{i}\right)$

The number of weeks (W) is the difference between the first week an area is considered affected and the last week of the season (end of November). Subsequently, we estimated the proportional impact of the alternative scenarios to the size of the blood donor population subject to blood safety measures using the formula:

$$\frac{Impact_{Scenario\;N}-Impact_{Baseline}}{Impact_{Baseline}}\;=\;proportional\;impact\;of\;WNV\;circulation\;on\;blood\;safety\;measures$$

We considered that the number of blood donations is proportional to the population and that the proportion of donations is constant across the affected areas [15]. The number of donations tested for WNV or deferral therefore increases proportionally to the number of affected areas and their respective population. We did not take into consideration movement of people between areas.

## Results

### Description of the overall epidemiological situation in European Union countries, 2013–2016

**Figure 1:**
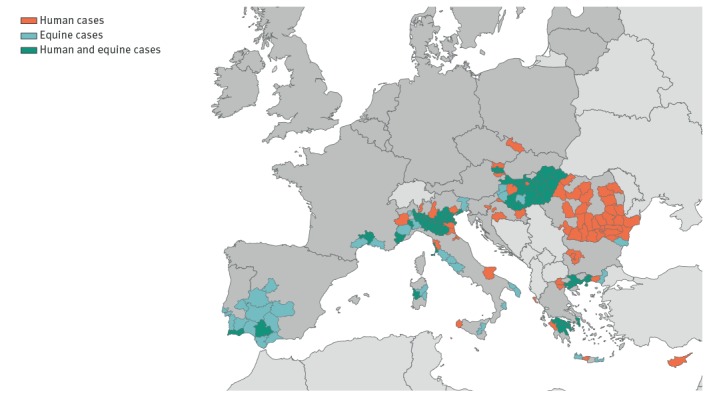
Distribution of human (n = 847) and equine (n = 553) West Nile virus infections in the European Union countries, 2013–2017, (n = 1,400)

**Figure 2:**
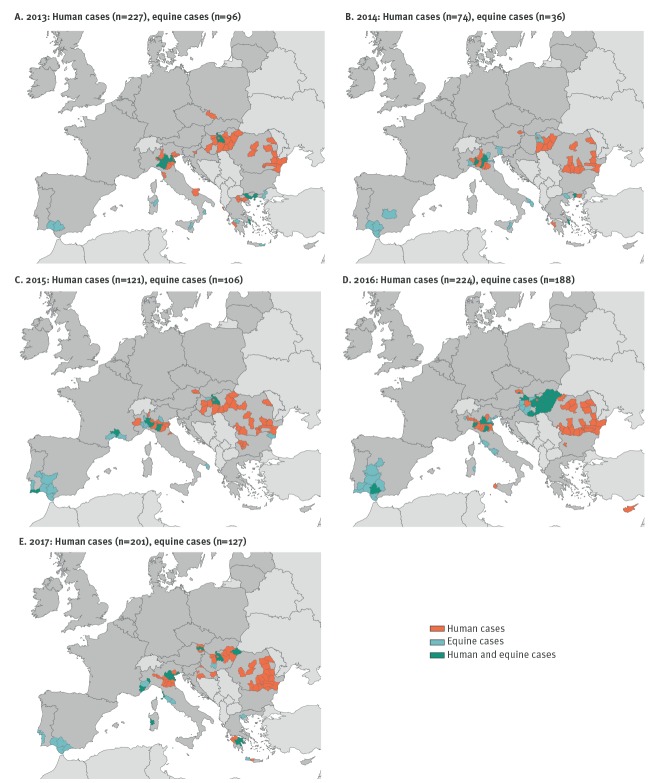
Geographical distribution of human and equine West Nile virus infections by NUTS-3 level and year, European Union countries, in: (A) 2013 (n = 323), (B) 2014 (n = 110), (C) 2015 (n = 227), (D) 2016 (n = 412), (E) 2017 (n = 328)

Between 2013 and 2017, 847 human WNV infections (yearly average: 170; range: 74–227) were reported by 13 EU countries. In the same period, 553 equine WNV infections were reported by eight EU countries (Table 1). Human cases were mainly reported in the south-eastern part of the EU whereas equine cases were reported in the south-east and south-west of the EU (Figures 1 and 2). Over the study period, WNV transmission occurred in 140 areas; human cases were reported in 109 areas and equine cases in 81 areas. Human and equine cases were frequently reported in the same areas during a given year. Of 13 countries with human cases, eight (Austria, Bulgaria, France, Greece, Hungary, Italy, Spain and Portugal) reported both human and equine cases, five countries (Croatia, Cyprus, the Czech Republic, Romania and Slovenia) reported human cases only. No country reported only equine cases (Figure 2).

**Table 1:** Number of non-imported human and equine cases of West Nile virus infections, European Union countries, 2013–2017 (n = 1,400)

Country	2013		2014		2015		2016		2017		Total	
	Human	Equine	Human	Equine	Human	Equine	Human	Equine	Human	Equine	Human	Equine
**Countries with human and equine cases**												
Austria	0	0	2	0	6	0	5	1	6	2	19	3
Bulgaria	0	0	0	0	2	1	2	0	1	0	5	1
France	0	0	0	0	1	41	0	0	2	1	3	42
Greece	86	15	15	4	0	0	0	0	48	13	149	32
Hungary	35	1	10	1	18	7	44	49	20	3	127	61
Italy	80	43	24	19	61	31	76	53	53	92	294	238
Portugal	–	0	–	0	1	9	0	6	0	3	1	18
Spain	0	37	0	12	0	17	3	79	0	13	3	158
**Countries with only human cases**												
Croatia	–	0	–	0	–	0	–	0	5	0	5	0
Cyprus	0	0	0	0	0	0	1	0	0	0	1	0
Czech Republic	1	0	0	0	0	0	0	0	0	0	1	0
Romania	24	0	23	0	32	0	93	0	66	0	238	0
Slovenia	1	0	0	0	0	0	0	0	0	0	1	0
**Total**	**227**	**96**	**74**	**36**	**121**	**106**	**224**	**188**	**201**	**127**	**847**	**553**

–: no report.

### Epidemiological situation in individual countries

The epidemiological situation in the different EU countries is heterogeneous [16].

#### Countries with human and equine cases

Austria and Bulgaria reported sporadic human and equine cases. In 2014, Austria reported its first human case and 2 years later its first equine case. Bulgaria reported human and equine cases in 2015 and human cases only in 2016 and 2017.

In France, WNV re-emerged in 2015. One human case and an outbreak among equids in the Camargue region in the south of France was reported. The first equine cases had disease onset 5 weeks before the human case. In 2017, another two cases were reported in the Alpes-Maritimes, an area where no human WNV infection had been reported before.

In 2013, Greece accounted for 38% (86/227) of the human cases and for 16% (15/96) of the equine cases reported in the EU. In 2014, the number of human cases in Greece dropped from the previous year’s 86 to only 15, and equine cases from 15 to only 4 cases, a decrease of 73%. In 2017, human and equine cases were reported again, after 2 consecutive years without any cases. Between 2013 and 2017, there were eight areas with both human and equine cases. In 2013, in one of the four areas with both human and equine cases, equine cases were reported before the first human case. In 2014 and 2017, all human cases were reported before equine cases.

**Figure 3:**
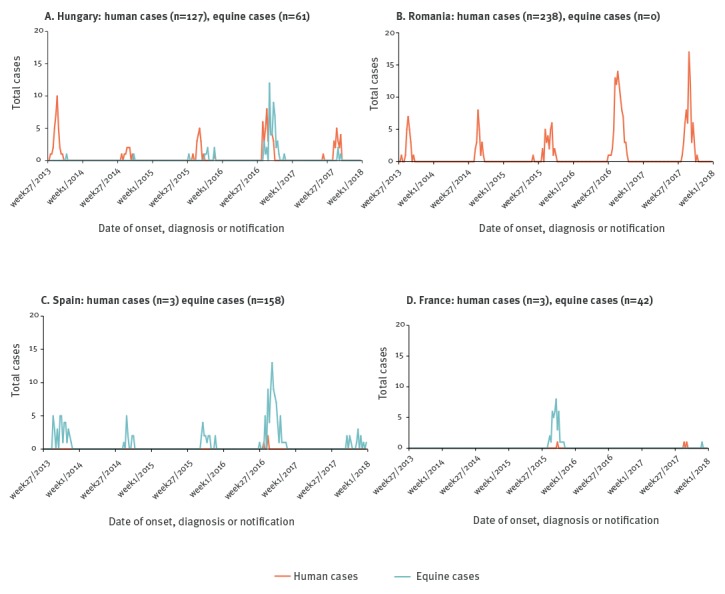
Weekly number of human and equine West Nile virus infections: (A) Hungary (n = 188), (B) Romania (n = 238), (C) Spain (n = 161), (D) France (n = 45), 2013–2017

Hungary reported 127 human and 61 equine cases, in 16 and 17 areas respectively, of 20 areas in total. The number of human and equine cases peaked in 2016 (Figure 3). In 2013, 2014 and 2017, most cases were localised in the eastern part of the country. In 2015 and 2016, the virus spread westwards. Between 2013 and 2017, 16 areas in Hungary reported both human and equine cases. Equine cases preceded human cases in two areas in 2016 and one in 2017, by 1.9, 3.7 and 0.3 weeks, respectively.

Italy reported 294 human and 238 equine cases between 2013 and 2017, which represent respectively 35% (294/847) and 43% (238/553) of all cases reported at the EU level. Human cases were reported in 29 areas in Italy and equine cases were reported in 32 areas, from a total of 110 areas. The number of human and equine cases in Italy was stable over the study period, except in 2014, when the number of human and equine cases dropped from 80 to 24 cases (70%); and from 43 to 19 cases, respectively, compared with 2013 (Table 1). Most of the human and equine cases (98% (289/294) and 95% (226/238), respectively) in Italy occurred in the northern part of the country, where the ecological conditions are most suitable for WNV transmission. In 13 of the total 32 affected areas in Italy, equine cases were reported in areas without human cases. Equine cases preceded the human cases in three areas in 2013, two in 2014, two in 2015 and four in 2017.

Portugal reported no human or equine cases in 2013 and 2014. One human case was reported in the south of the country in 2015. In 2015, 2016 and 2017 Portugal reported 18 equine cases in Alentejo, Algarve and the Área Metropolitana de Lisboa, all in the south of the country. 

Between 2013 and 2017, Spain reported equine cases every year, with peaks in 2013 and 2016, but human cases only in 2016. A total of 158 equine cases were detected, representing 29% (158/553) of the EU cases. All human cases occurred in one area, whereas equine cases were reported in nine areas. Most equine cases and all human cases were reported in Andalucía, in the south-west of Spain. During the study period, the disease in equids spread from the far south towards more central areas in Spain (e.g. Ávila). In 2016, human and equine cases were reported in Sevilla, and human cases preceded equine cases by 1 week.

#### Countries with only human cases

Croatia reported five human cases in 2017 in the northern part of the country.

Cyprus reported its first human WNV infection in 2016.

The Czech Republic reported one human case in 2013 and no cases thereafter.

In Romania, 238 human cases were reported between 2013 and 2017 in 29 of the 42 areas. On average 48 human cases (ranging from 23 and 93) were yearly reported with a peak in 2016.

Slovenia reported one human case in 2013 and no cases after that.

### Consequences of using equine and human cases to define an affected area

Between 2013 and 2017, the annual number of areas (range) with exclusively human cases or with exclusively equine cases or with both human and equine cases was on average 38 (range: 29–47), 13 (range: 8–19), and 6 (range: 2–17), respectively (Table 2). Human and equine cases were reported in the same areas on 61 occasions; either equine case(s) preceded human case(s) (n = 16; 26%) or equine case(s) followed human case(s) (n = 45; 74%).

**Table 2:** Overview of affected areas with human, equine or human and equine cases of West Nile virus infection in the European Union, 2013–2017

	Areas with human cases only					Areas with equine cases only					Areas with both human and equine cases									
											Equine cases before human cases					Equine cases after human cases				
	2013	2014	2015	2016	2017	2013	2014	2015	2016	2017	2013	2014	2015	2016	2017	2013	2014	2015	2016	2017
**Countries with human and equine cases**																				
Austria	NC	2	2	1	2	NC	NC	NC	NC	NC	NC	NC	NC	NC	NC	NC	NC	NC	1	1
Bulgaria	NC	NC	2	1	1	NC	NC	1	NC	NC	NC	NC	NC	NC	NC	NC	NC	NC	NC	NC
France	NC	NC	NC	NC	NC	NC	NC	2	NC	NC	NC	NC	1	NC	NC	NC	NC	NC	NC	1
Greece	8	2	NC	NC	3	3	1	NC	NC	2	1	NC	NC	NC	NC	3	2	NC	NC	2
Hungary	11	6	9	2	7	NC	1	1	4	1	NC	NC	NC	2	1	1	NC	1	10	1
Italy	8	6	10	11	10	3	7	5	7	3	3	2	2	NC	4	5	3	3	5	4
Portugal	NC	NC	NC	NC	NC	NC	NC	2	2	2	NC	NC	NC	NC	NS	NC	NC	1	NC	NC
Spain	NC	NC	NC	NC	NC	2	4	4	6	4	NC	NC	NC	NC	NC	NC	NC	NC	1	NC
**Countries with only human cases**																				
Croatia	NC	NC	NC	NC	4	NC	NC	NC	NC	NC	NC	NC	NC	NC	NC	NC	NC	NC	NC	NC
Cyprus	NC	NC	NC	1	NC	NC	NC	NC	NC	NC	NC	NC	NC	NC	NC	NC	NC	NC	NC	NC
Czech Republic	1	NC	NC	NC	NC	NC	NC	NC	NC	NC	NC	NC	NC	NC	NC	NC	NC	NC	NC	NC
Romania	11	13	14	22	20	NC	NC	NC	NC	NC	NC	NC	NC	NC	NC	NC	NC	NC	NC	NC
Slovenia	1	NC	NC	NC	NC	NC	NC	NC	NC	NC	NC	NC	NC	NC	NC	NC	NC	NC	NC	NC
**Total**	**40**	**29**	**37**	**38**	**47**	**8**	**13**	**15**	**19**	**12**	**4**	**2**	**3**	**2**	**5**	**9**	**5**	**5**	**17**	**9**

NC: no cases.

In the first scenario, where both human and equine case(s) define an affected area, 76 areas would have been considered affected in 2016 and 73 in 2017 (Table 3). For instance, the start of the season in Cádiz, Spain, was defined by an equine case in week 27 in 2016. We estimated that in this scenario an increase of 34% in 2016 and 13% in 2017 of blood safety measures (including blood testing or deferral) is necessary compared to the baseline scenario based on human cases only.

**Table 3:** Increase in impact of blood safety measures based on the use of equine cases of West Nile virus infection to define an affected area, European Union, 2016 and 2017

	Reference		Scenario 1				Scenario 2			
	An affected area is defined based on human cases		Both human and equine cases define an affected area				In addition to human cases, equine cases define an affected area if at least one human case was detected in that area in one of the 3 previous years for 2016 and 4 previous years for 2017			
	Number of affected areas (increase in impact on blood safety measures)									
	2016	2017	2016		2017		2016		2017	
	n	n	n	Impact	n	Impact	n	Impact	n	Impact
**Countries with human and equine cases**										
Austria	2	3	2	0%	3	0%	2	0%	3	0%
Bulgaria	1	1	1	0%	1	0%	1	0%	1	0%
France	0	1	0	0%	1	0%	0	0%	1	0%
Greece	0	5	0	0%	7	122%	0	0%	6	106%
Hungary	14	9	18	9%	10	3%	16	6%	9	0%
Italy	16	18	23	79%	21	12%	18	15%	18	4%
Portugal	0	0	2	NA	2	NA	1	NA	0	0%
Spain	1	0	7	143%	4	NA	1	0%	1	NA
**Countries with only human cases**										
Croatia	0	4	0	0%	4	0%	0	0%	4	0%
Cyprus	1	0	1	0%	0	0%	1	0%	0	0%
Czech Republic	0	0	0	0%	0	0%	0	0%	0	0%
Romania	22	20	22	0%	20	0%	22	0%	20	0%
Slovenia	0	0	0	0%	0	0%	0	0%	0	0%
**Total**	**57**	**61**	**76**	34%	**73**	13%	**62**	7%	**63**	9%

NA: the impact on blood safety cannot be expressed as a percentage increase as there was no affected area in the reference period.

The impact was calculated as a proportional increase of blood safety measures if equine cases were to be used in addition to human cases for defining an affected area, compared to the baseline scenario based on human cases only.

In the second scenario, we estimated that an increase of 7% in 2016 and 9% in 2017 of blood safety measures is necessary compared to the baseline scenario in 2016 and by 9% in 2017.

The impact on blood safety cannot be expressed as a percentage increase in areas where there was no affected area in the reference period (Table 3). However, for example, in Portugal in 2016, only around 1% of the population per week of the overall population of potential donors would need to be covered by blood safety measures under scenario 2.

## Discussion

This study confirms that the distribution patterns of WNV in the EU are extremely heterogeneous, ranging from countries with no human or equine cases to countries where the disease is endemic in human and equids. The diversity in the epidemiological situation of WNV infections between humans and equids is likely due to a series of factors such as the difference in immunity and susceptibility of certain human (e.g. elderly and immunocompromised) [17] and equine populations, the different local vector feeding behaviour and vector abundance and the differences in the sensitivity of surveillance systems [18]. The occurrence of cases may also be influenced by the variability of the human or equine population densities, with more cases expected in areas with higher densities and the differences in exposure to mosquitoes with equids often being more exposed to mosquito bites as they are generally kept outside, especially during summer. Although areas with human and equine cases highlight the circulation of the virus, it should be noted that the absence of cases does not confirm the absence of the virus in an area.

Since a large proportion of human and equine infections remain asymptomatic, surveillance as currently performed in the EU only captures the tip of the iceberg. Romania, for instance, has not reported any equine case to ADNS as they did not identify any symptomatic equids that are, according to the case definition, the only cases that must be notified. One of the hypotheses raised for the absence of clinical equine cases is the possible humoral immunity of equids in Romania developed through natural exposure to WNV over the years [19].

The sensitivity of equine surveillance may be influenced by the use of equine vaccines and equids being more seroprevalent, which reduces the susceptible horse population and limits the use of seroepidemiological studies [20]. In practice, however, equine vaccination is rarely conducted in the EU as the vaccine is effective for 1 year only and vaccination is often at the owner’s expenses [4,21]. National vaccination numbers for each country are not available.

Climatic and environmental conditions play a role in the suitability for WNV circulation. For example, in affected areas in the Camargue region in France, multiple favourable conditions are known to support WNV amplification cycles including large natural reserves, extensive wetlands, substantial drainage and irrigation efforts, and lack of adult mosquito controls [21]. These conditions directly influence the abundance of potential vectors and the presence of amplifying birds creating high risk areas for WNV circulation. In northern Italy, a significantly higher abundance of the vector was recorded in warmer and less rainy conditions [22]. These conditions cause virus spill over outside the sylvatic cycle, to humans and/or equids.

Surveillance of WNV infections only capture a small proportion of the cases, but considering that this was constant during the study period, this has not been considered as a limitation. The estimation of the impact on blood safety measures under the alternative scenarios assumes that donation practices are proportional to the population, which may not be the case, in particular if the demographic structure of the population varies across affected areas. In addition, as travellers who visited affected areas are not taken into account, the real impact of WNV circulation on blood safety measures according to the alternative scenarios are underestimated. The impact of the definition of affected areas on blood safety measures will not only touch affected countries but also EU countries that have never experienced WNV circulation (such as the Netherlands or Belgium).

There is no ‘one size fits all’ surveillance strategy applicable in the EU [16] and each country should adapt its surveillance to its epidemiological situation, its surveillance objectives and its capacity. Italy implemented a fully integrated surveillance system with surveillance in humans, equids, mosquitoes and birds, and findings of animal and vector or of human infections triggers the screening of blood donors in the affected areas [23]. Although mosquito surveillance is considered effective, as virus detection in mosquitoes often precedes human cases [22], this surveillance strategy is costly. In 2010, Spain stopped its routine WNV entomological surveillance due to insufficient cost-effectiveness [24]. Dead-bird surveillance is generally not considered useful in Europe as bird mortality due to WNV infection is rare in most European countries [25]. Although equine surveillance is generally less predictive, it is considered cheaper than mosquito and bird surveillance. Austria, France, Greece, Hungary, Italy and Spain have implemented equine surveillance and public health authorities are receiving awareness alerts following the detection of equine cases [16,23,26].

The analysis of TESSy and ADNS data found no evidence that presence of equine cases in a given area would increase the likelihood of human cases occurrence in the same area. In Spain for instance, equine cases have been reported repeatedly in multiple areas in Andalucía, where no human cases have ever been reported to date. In Italy, veterinary surveillance identified equine cases with neurological symptoms in areas without any human cases (e.g. in Sardinia and Tuscany). A similar situation occurred in Greece where equine cases were reported in areas such as Lasithi without any human cases.

TESSy and ADNS datasets represent only a small fraction of the human and equine infections and do not provide the full extent of the temporal and geographical distribution of the virus. Over time, equine population surveillance in areas with known virus circulation would become less effective as herd immunity is expected to increase. To assess any correlation between human and equine cases, coordinated seroprevalence studies in humans and equids would be necessary.

Adding equine cases to the definition of an affected area would increase the number of affected areas and consequently would increase the population subject to blood testing or blood deferral. Should an equine case be treated as an equivalent to a human case, the impact on blood safety measures would be major, without current evidence of an association between equine and human cases. Using equine cases as a trigger only in those areas where human cases have been reported in the past would increase the timeliness of blood safety measures with only a limited impact on the costs. Despite the absence of evidence that occurrence of an equine case increases the likelihood of occurrence of human cases, the detection of an equine case does indicate an ongoing epizootic transmission, placing humans at risk.

Knowledge of preferences in species involved in transmission to humans and equids and knowledge on the conditions for the spill-over of the virus to humans and equids are crucial in providing early warnings for public health [27]. Well-established routine veterinary (e.g. in birds) and entomological surveillance is essential to alert for human WNV infections, especially in areas suitable for WNV circulation. Correlation studies should be based on both symptomatic and asymptomatic cases. Cost-effectiveness studies will be needed to estimate the impact of the costs on blood safety measures in areas were equine cases are used as a trigger to implement these measures. As part of the cost-effectiveness studies, options to implement de-triggering criteria of blood safety measures should be also investigated. Besides the two alternative scenarios proposed in this paper, other options could be investigated.

In the United States, all blood donations are tested during WNV transmission season and the results of the blood donations are used to estimate the actual frequency of the disease [15]. Although in the EU not all blood donors are systematically screened for WNV infections, information on the number of positive cases among blood donors could contribute to assess the correlation between human and equine cases, by exploring whether there are asymptomatic human cases in areas with equine cases, where no clinical human cases have been reported.

During WNV transmission season both human and animal health authorities in the EU provide data in a timely manner, allowing a close-to-real-time assessment of the epidemiological situation. Data on equine cases collected through ADNS are crucial to complement TESSy data in order to raise awareness among public health experts, and to enhance passive or active surveillance among humans living or having visited areas with equine cases [21,28]. For this purpose, ECDC has been publishing weekly epidemiological reports presenting the distribution of human and equine cases reported to TESSy and ADNS since 2017 [29].

### Conclusions

To our knowledge, this is the first study that co-analyses human and equine WNV surveillance data at the EU level. Although we found no evidence of an association between equine and human cases, using equine cases as a trigger only in those areas where human cases have been reported in the past could increase the timeliness of blood safety measures with only a limited impact on the costs. Further studies are needed to investigate the extent to which equine data can be used as a trigger for the start of blood safety measures. In the meanwhile, equine WNV data could already be used to raise awareness among public and veterinary health experts and to trigger an enhanced surveillance and prevention activities.

## European Union West Nile fever working group

Stephan Aberle (Medical University Vienna, Austria), Friedrich Schmoll (Austrian Agency for Health and Food Safety, Austria), Iva Christova (National Center of Infectious and Parasitic Diseases, Bulgaria), Simona Chakarova (National Diagnostic and Research Veterinary Institute, Bulgaria), Iva Pem Novosel, (Croatian Institute of Public Health, Croatia), Maria Koliou (Ministry of Health, Cyprus), Bohumir Kriz (National Institute of Public Health, Czech Republic), Marie Claire Paty (Agence Nationale de Santé Publique, France), Sylvie Lecollinet (ANSES, Laboratoire de Santé Animale de Maisons-Alfort, France), Danai Pervanidou (National Public Health Organization – Greece/ former Hellenic Center for Disease Control & Prevention, Greece), Sotiria-Eleni Antoniou (Department of Infectious and Parasitic Diseases, Ministry of Rural Development and Food, Greece), Orsolya Nagy (National Public Health Institute, Hungary), Eszter Mezei (Ministry of Human Capacities, Hungary), Péter Malik (Veterinary Diagnostic Directorate, Hungary), Zsolt Terjék (National Food Chain Safety Office, Hungary), Caterina Rizzo (Bambino Gesù Children's Hospital, IRCCS, Rome, Italy), Paolo Calistri (Istituto Zooprofilattico Sperimentale dell’Abruzzo e del Molise “G. Caporale”, Italy), Sofia Quintans (Division of Epidemiology and Animal Health from the Directorate-General of Food and Veterinary, Portugal), Anca Sirbu (National Centre for Communicable Diseases Surveillance and Control, Romania), Dudu Florentina (National Sanitary Veterinary and Food Safety Authority, Romania), Doru Hristescu (Institute for Diagnosis and Animal Health, Romania), Inés Moreno Gil (Ministry of Agriculture, Food and Environment, Spain), Rubén Villalba Martínez (Ministry of Agriculture, Food and Environment, Spain), María Paz Sanchez-Seco (Institute of Health Carlos III, ISCIII, Spain), María José Sierra (Ministry of Health, Consumer Affairs and Social Welfare, Spain), Maja Socan (National Institute of Public Health (NIJZ), Slovenia).
